# Synthesis of multidentate ligands with amido or amino donor groups for the preparation of rhenium and technetium radiopharmaceuticals

**DOI:** 10.1007/s10967-012-2356-z

**Published:** 2012-12-11

**Authors:** S. M. D. Al-Nuzal, H. M. A.-K. Al-Azzawi, Z. M. J. Al-Mosawy

**Affiliations:** 1 Environmental Research Centre, The University of Technology, Baghdad, Iraq; 2 Directorate of Chemistry and Petrochemical Industry, Ministry of Science and Technology, Al-Jadiryia, Baghdad, Iraq

**Keywords:** Multidentate ligands, Rhenium and technetium complex ReOCl_3_(PPh_3_)_2_, ReOCl_3_L, Labeled with ^99m^Tc pertechnetate, Radioactive purity, Radiopharmaceuticals

## Abstract

A new method to prepare novel semi-rigid multidentate ligands containing nitrogen atom, to coordinate with rhenium and technetium, was established. The method was based on formylation of substituted anilines, followed by Mannich reaction with glycine and paraformaldehyde. The method was very promising to design ligands of various molecular structures (L_1_–L_5_) to coordinate with rhenium metal ions. The complexes were prepared through ligand exchange with the complex ReOCl_3_(PPh_3_)_2_, giving new complex of the structure ReOCl_3_L_(1–5)_. The prepared ligands and complexes were identified by the use of UV–vis, and infrared absorption spectrometric techniques, elemental analysis, molecular weight determination by depression of freezing point. These ligands were labeled with ^99m^Tc pertechnetate, and the labeling efficiency of the complexes was measured using a well type scintillation gamma counter equipment and obtained a good yield.

## Introduction

The coordination chemistry of technetium has rapidly developed, owing to its short half-life, pure photon emission, and suitable energy of ^99m^Tc, make it the best choice for imaging studies [1–3]. The more recent introduction of β-emitting isotopes ^188^Re and ^186^Re in diagnostic imaging and radiotherapy boost the chemistry of rhenium as well [4–7]. A great number of chelate ligands for the encapsulation of rhenium and technetium have been prepared in the search of novel, selective, and effective agents for radiodiagnostic imaging and therapy.

Among the first of these ligands is that containing the peptide bonds of glycine and other amino acid derivatives in various molecular design, which were commonly used for imaging of the hepatobiliary system. There are three ^99m^Tc-HIDA (2,6-dimethylphenylcarbamoylmethyl)iminodiacetic acid) analogues which have been approved for this purpose; ^99m^Tc-Lidofenin, ^99m^Tc-Mebrofenin, ^99m^Tc-Disofenin, and *N*-(2-pyridylmethyl)iminodiacetic acid. The lipophilic properties of this compound were demonstrated in chloroform extraction studies where more than 80 % of the ^99m^Tc-ligands were extracted into the organic phase from the aqueous phase. The exact nature of the complexes is uncertain but it was proposed to contain two ligands coordinated in an octahedral configuration and bear a single negative charge [8, 9]. Other type of ligands consists of small peptides of glycine and other amino acids, which have proved successful in sequestering these metals.

An example is diethylenetriaminepentaacetic acid (DTPA), mercaptoacetyl triglycerine (known as MAG-3 in the market) etc. The labeling of antibodies with ^188^Rh using MAG-3 as a bifunctional chelating agent has been optimized and automated [10–16]. Variety of monodentate ligands can be combined with tetradentate Schiff-base ligands to give mixed-ligand rhenium complexes, such as N_2_O_2_-calix[4]arene Rhenium Complexes [17].

The present work will focus on the development of a new and simple synthetic procedure of new amino acid (glycine) chelates combined with an aniline substituted moiety through carbamoyl group for labeling with rhenium and technetium metals. Briefly, this study related to their ability to coordinate to rhenium and technetium has shown their potential of using them as new imaging probes.

## Experimental

### Chemicals and instruments

Substituted anilines (*p*-aminobenzoic acid and 2-aminopyrimidine) were purchased from BDH; 4-chloro-2-nitroaniline from Merck) diphenyl amine, and phenylene diamine from Fluka. Formaldehyde and rhenium metal powder purchased from Aldrich, glycine from Riedel de Häen. Melting points were measured with electrothermal melting Point (BÜCHI 535). UV–visible spectra were obtained with Shimadzu UV–Visible double beam scanning Spectrophotometer-260. Infrared spectrophotometric spectra were obtained Pye-Unicom-SP3-100-spectrophotometer with KBr disc. Perkin Elmer CHN Elemental Analyzer was used for elemental analyses. Radioactivities were measured by using a well type scintillation gamma counter equipment (berthold MAG 312 West–Germany).

### General formylation procedure

A mixture of (0.030 mol) of substituted aniline and formic acid (10.0 mL) was refluxed for 8 h. Formic acid was removed by evaporation, and the residue was left over filter paper for 1 h. The residue was transferred a beaker of 100 mL, washed with 10.0 mL distilled water, and then left over watch glass to dry at room temperature.

### General Mannich reaction

A mixture of a formyl derivative of substituted aniline (0.006 mol), paraformaldehyde (0.18 g, 0.006 mol), glycine (0.46 g, 0.006 mol), distilled water (10.0 mL) and 95 % ethanol (25.0 mL) in 100 ml r.b.f, was refluxed for 10 h. The mixture was left to cool, filtered, and then washed with distilled water (20.0 mL). The precipitate was dried at 50 °C overnight, to give the derivatives (L1, L2, L3, L4, and L5).

### Preparation of the complexes ReOCl_3_(PPh_3_)_2_

Rhenium metal powder (0.5 g, 2.0 mmol) was gradually treated with 9.0 mL of 35 % hydrogen peroxide in ice bath. The ice bath was replaced with water bath and the solvent was evaporated to 1–2 mL solution. The ice bath was replaced again, and then added with stirring a solution mixture of 5.0 mL concentrated hydrochloric acid and triphenyl phosphine (PPh_3_, 5.0 g, 1.0 mmol) in acetone (25.0 mL). When a yellowish green precipitate was formed. The mixture was stored to for reaching room temperature for 1 h and filtered. The precipitate was washed with 10.0 mL ethanol and dried at room temperature (2.2 g, 96 %, and mp 213 °C) [18].

### Preparation of the complexes ReOCl_3_L

An amount of the complex ReOCl_3_(PPh_3_)_2_ (0.20 g, 0.04 mmol) was placed in 100.0 mL r.b.f, and treated with a mixture of the ligand (0.40 mmol) and 95 % ethanol (2.0 mL). The mixture was refluxed for 90 min and color changement was observed. The flask was cooled and the precipitate was filtered with filter paper and then, dried at room temperature overnight.

### Radiochemical purity

For labeling, ligand solution (0.20 mg in 0.4 mL of saline solution) was mixed with freshly prepared solution of hydrated stannous chloride (containing 0.30 mg SnCl_2_·2H_2_O in 0.20 mL of 0.2 N HCl). The resulting mixture was labeled by adding a suitable volume 2.0–5.0 mL of ^99m^Tc-pertechnetate (0.5–10 mCi) eluate from ^99^Mo to ^99m^Tc generator (CIS–biointernational, France). Radiochemical labeling analysis was performed by adding a suitable volume (0.10–0.30 mL) of the above labeled preparation on the top of a column (1 × 20 cm) packed with Sephadex-25-fine (Pharmacia, Sweden). The column was eluted with normal saline solution, and (3.0 mL) fractions were collected and the radioactivity of each fraction was counted with a well type scintillation counter to obtain the labeling efficiency of each ligand.

## Results and discussion

**Fig. 1:**
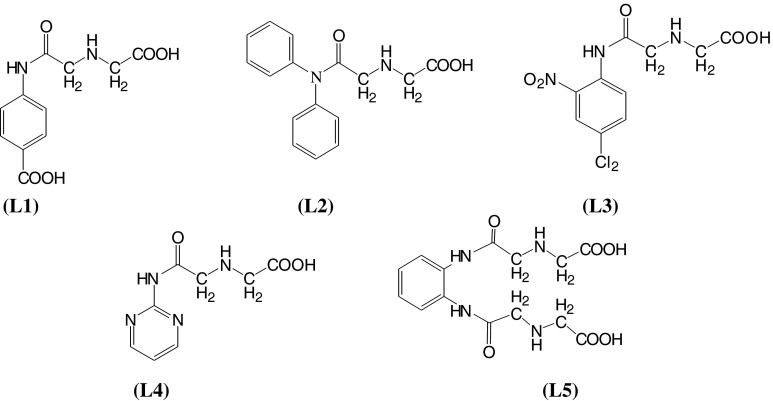
Chemical structure of the new ligands; (L1) *N*-Glycylacetyl *p*-aminobenzoic acid, (L2) *N*-Glycylacetyl diphenylamine, (L3) *N*-Glycylacetyl 4-chloro-2-nitroaniline, (L4) *N*-Glycylacetyl 2-pyrimidine, and (L5) *Bis*(*N*-Glycylacetyl) phenylene diamine

**Fig. 2:**
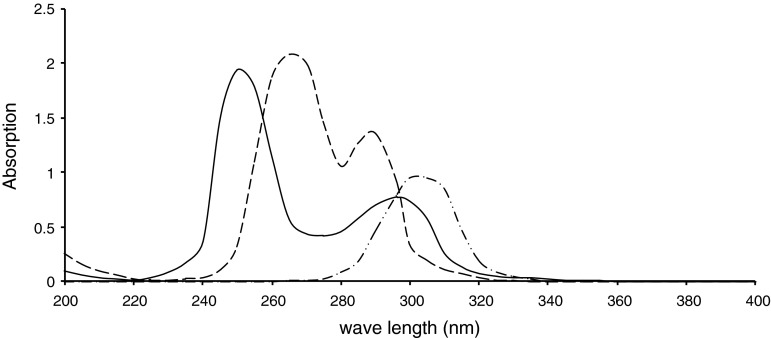
The UV–visible spectrum of *p*-amino benzoic acid (*dashed line*), *N*-formyl *p*-amino benzoic acid (*dot-dashed line*), and *N*-Glycylacetyl *p*-aminobenzoic acid (*continuous line*)

**Fig. 3:**
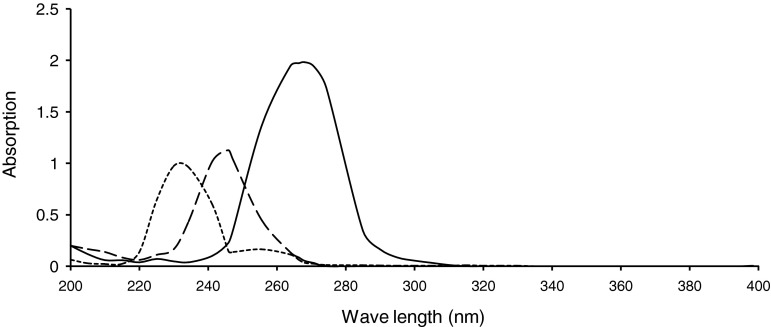
The UV–visible spectrum of diphenyl amine (*dashed line*), *N*-formyl diphenyl amine (*dot-dashed line*), and *N*-Glycylacetyl diphenylamine (*continuous line*)

**Fig. 4:**
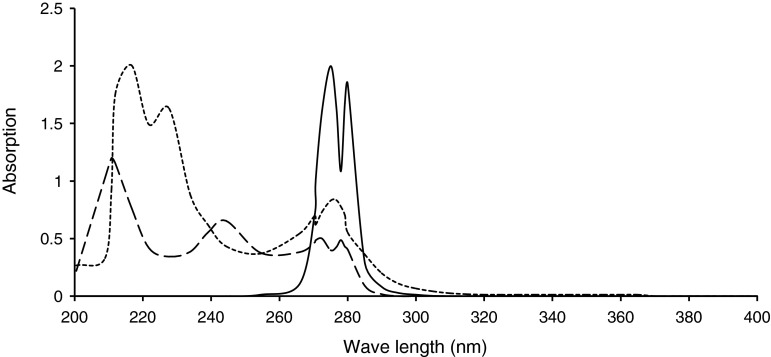
The UV–visible spectrum of phenylene diamine (*dashed line*), *N*,*N*′-diformyl phenylene diamine (*dot-dashed line*), and Bis-*N,N′*(Glycylacetyl) phenylene diamine (*continuous line*)

The formyl derivatives and Mannich reaction substituted anilines were prepared following the general procedures mentioned in the experimental part. They were obtained in good purity and radiolabeling yields (~70 % in general). Their physical, UV–visible, and IR absorption spectroscopic properties of the formyl derivatives (I–V) and their Mannich reaction products with glycine (L1–L5) as well as the 1:1 coordination products ReOCl3L (C1–C5), were presented in Tables 1, 2, and 3. The proposed chemical structure of the Mannich reaction products were presented in Fig. 1. These results were in good agreement with the proposed chemical structure of the products. All formyl derivatives showed two new absorption bands at 1,668–1,735 and at 2850–2750 cm^−1^ in the FT-IR spectra corresponding the attachment of formyl group on anilines. The first one was due to the C=O stretching, while the second one was due to the C–H aliphatic stretching. The second absorption band disappeared upon Mannich reaction substitution. *λ*_max_ of the UV–visible absorption spectra of the substituted anilines used as starting materials showed clear shift to higher wave length upon substitution with the formyl group, and with methyl glycine after Mannich reaction. Generally, this shift is accompanied with increase in the value of *λ*_max_ of the products due to the hyperconjugation of the amine proton with benzene ring (Figs. 2, 3, 4). This new method will offer reliable procedure to design ligands of the following general structure.

**Table 1:** The physical properties of the formyl derivatives (I–V) and their Mannich reaction products with glycine (L1–L5)

	Chemical formula	M.wt		m. p (°C)	UV–visible		Color	Yield %
		(theor.)	(meas.)^a^		*λ*_max_ (nm)	*ε* (l mol^−1^)		
I	C_8_H_7_NO_3_	165	166.5	225–227	296	4,256	Purple	70
II	C_13_H_11_NO	197	196	70–73	246	9,700	Gray	69
III	C_7_H_5_N_2_O_3_Cl	200	198	142–145	247	12,750	Yellow	73
					362	2,425		
IV	C_5_H_5_N_3_O	123	122	166–169	232	16,000	Brown	71
					271	2,250		
V	C_8_H_8_N_2_O_2_	164	163	170–174	208	11,400	Purple	76
					244	6,100		
					250	14,992		
					272	5,600		
					279	5,600		
L1	C_11_H_12_N_2_O_5_	252.22	250.2	171–175	296	4,256	Brown	70
					290	4,400		
L2	C_16_H_16_N_2_O_3_	284	287.5	67–70	246	9,700	Brown	69
L3	C_10_H_10_N_3_O_5_Cl	287.66	289	136–139	266	9,200	Orange	73
					348	3,400		
L4	C_8_H_10_N_4_O_3_	210.19	212	151–153	297	2,950	Brown	71
L5	C_14_H_18_N_4_O_6_	338.32	342	162–165	264	5,657	Purple	76
					280	5,200		

^a^By depression of freezing point

**Table 2:** The elemental analysis of the formyl derivatives (I–V), Mannich reaction products with glycine (L1–L5), and the 1:1 coordination products ReOCl_3_L (C1–C5)

	Chemical formula	M. wt	Carbon		Hydrogen		Nitrogen	
			Theo.	Det.	Theo.	Det.	Theo.	Det.
I	C_8_H_7_NO_3_	165.15	58.18	58.00	5.62	4.10	8.48	8.50
II	C_13_H_11_NO	197.23	79.16	57.20	5.62	5.50	7.10	7.20
III	C_7_H_5_ClN_2_O_3_	206.58	41.92	42.0	2.51	2.40	13.97	14.00
IV	C_6_H_5_N_3_O_3_	167.12	43.12	42.95	3.02	2.95	25.14	26.20
V	C_8_H_8_N_2_O_2_	164.16	58.53	58.40	4.91	4.90	17.06	17.10
L1	C_11_H_12_N_2_O_5_	252.22	52.38	52.35	4.80	4.65	11.11	11.30
L2	C_16_H_16_N_2_O_3_	284.31	67.59	67.62	5.67	5.50	9.85	10.20
L3	C_10_H_10_ClN_3_O_5_	287.66	41.75	41.55	3.50	3.40	14.61	14.90
L4	C_8_H_10_N_4_O_3_	210.19	45.71	45.55	4.80	4.75	26.66	26.60
L5	C_14_H_18_N_2_O_6_	338.32	49.70	49.50	5.36	5.25	16.56	16.55
C1	C_11_H_12_N_2_O_6_Cl_3_Re	592.87	23.56	23.40	2.16	2.05	4.85	5.00
C2	C_16_H_16_N_2_O_4_Cl_3_Re	592.7	32.41	31.95	2.72	2.60	4.73	4.60
C3	C_10_H_10_N_3_O_6_Cl_4_Re	594.9	20.14	31.88	1.69	1.65	7.05	6.98
C4	C_8_H_10_N_4_O_4_Cl_3_Re	518.8	18.52	18.40	1.94	1.85	10.80	10.50
C5	C_14_H_18_N_4_O_7_Cl_3_Re	646.9	25.99	25.85	2.80	2.90	8.66	8.70

**Table 3:** The IR absorption band of the formyl derivatives, Mannich reaction products with glycine, and the 1:1 coordination products ReOCl_3_L

	O–H Stretch.	N–H Stretch.	N–H Stretch.	C=C Stretch.	C–N Aliphatic Stretch.	C–H Aliphatic Stretch.	C–H Aromatic O.O.P	N–H Bend	N–H O.O.P	Others N=O
I	2,507	3,311	1,668	1,556	1,170	2798	665	1,490	806	–
	3,400			1,610						
II	–	–	1,672	1,589	1,180	2800	692	1,490	842	–
III	–	3,282	1,679	1,571	1,149	2922	646	1,502	788	1,342
			1,614							
IV	–	3,344	1,710	1,523	1,112	2790	640	1,454	804	1,502
			1,652							
V	–	3,398	1,735	1,569	1,132	2781	710	1,569	777	–
L1	2,500	3,309	1,697	1,562	1,172	–	607	1,498	812	–
	3,400			1,608						
L2	2,341	3,108	1,672	1,593	1,182	–	611	1,492	844	–
	3,350									
L3	2,611	3,182	1,679	1,573	1,151	–	648	1,500	796	1,340
	3,367									
L4	2,374	3,560	1,695	1,525	1,118	–	640	1,454	804	–
	3,442		1,595							
L5	2,542	3,210	1,740	1,506	1,130	–	702	1,506	746	–
	3,450			1,612

**Fig. 5:**
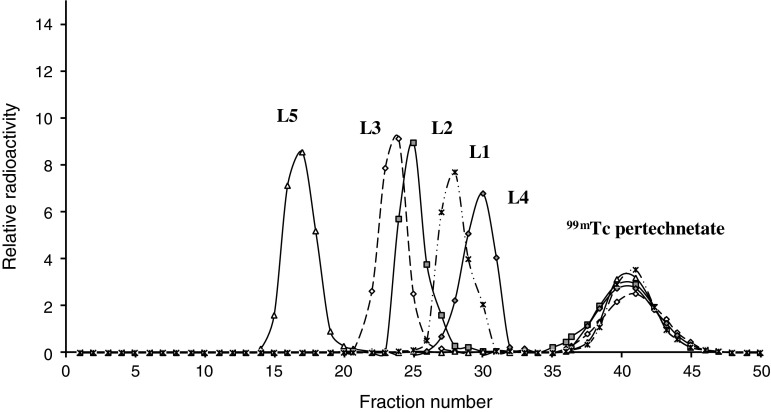
Chromatography separation profile of the labeled ligands on a Sephadex G-25 column of ^99m^Tc pertechnetate labeling with (L1) *N*-Glycylacetyl p-aminobenzoic acid, (L2) with *N*-Glycylacetyl diphenylamine,(L3) *N*-Glycylacetyl 4-chloro-2-nitroaniline, (L4) with *N*-Glycylacetyl 2-pyrimidine, and (L5) with *Bis*(*N*-Glycylacetyl) phenylene diamine

This structure will contain a lipophilic part of aromatic nucleus, and the hydrophilic part which can be any other amino acids. Rhenium complexes of these complexes were prepared by ligand substitution with the rhenium complex, oxotrichloro(triphenyl phosphine)rhenium(V) [ReOCl_3_(PPh_3_)_2_] with 1:1 mol ratio of the metal:ligand. Chromatography profile of the labeled ligands on a Sephadex G-25 column shows that high percentage of the radioactivity was recovered in the void volume associated with the ligand fraction (Fig. 5). It gives good indication about the efficiency of labeling these ligands with Na^99m^TcO4. Future work will be directed towards the direct application of these ligands in radiopharmaceutical imaging.
